# Alternatives to Antimicrobial Treatment in Bovine Mastitis Therapy: A Review

**DOI:** 10.3390/antibiotics12040683

**Published:** 2023-03-30

**Authors:** Dragana Tomanić, Marko Samardžija, Zorana Kovačević

**Affiliations:** 1 Department of Veterinary Medicine, Faculty of Agriculture, University of Novi Sad, Trg Dositeja Obradovica 8, 21000 Novi Sad, Serbia; 2 Faculty of Veterinary Medicine, University of Zagreb, Heinzelova 55, 10000 Zagreb, Croatia

**Keywords:** alternative treatment, antimicrobial stewardship, dairy cows, mastitis

## Abstract

Despite preventive and therapeutic measures, mastitis continues to be the most prevalent health problem in dairy herds. Considering the risks associated with antibiotic therapy, such as compromised effectiveness due to the emergence of resistant bacteria, food safety issues, and environmental impact, an increasing number of scientific studies have referred to the new therapeutic procedures that could serve as alternatives to conventional therapy. Therefore, the aim of this review was to provide insight into the currently available literature data in the investigation of non-antibiotic alternative approaches. In general, a vast number of in vitro and in vivo available data offer the comprehension of novel, effective, and safe agents with the potential to reduce the current use of antibiotics and increase animal productivity and environmental protection. Constant progress in this field could overcome treatment difficulties associated with bovine mastitis and considerable global pressure being applied on reducing antimicrobial therapy in animals.

## 1. Introduction

Mastitis is one of the health disorders that is common in dairy herds, affecting production, animal health, welfare, and the economy of the industry worldwide [1,2]. The high incidence of mastitis is directly related to increased milk production [3], with an estimation that this disease affects between 15 and 20% of the dairy cow population each year [4]. Being known as a multifactorial disease, its incidence depends on pathogens, udder defence mechanisms, and the presence of environmental factors [5,6]. It is defined as inflammation of the mammary gland caused by many different bacteria strains [7], as well as fungi such as *Candida* spp. [8] and algae such as *Prototheca* [9]. Literature data show that more than 140 microorganisms are associated with the aetiology of mastitis, indicating that the most common mastitis-associated bacteria include *Staphylococcus aureus*, *Streptococcus agalactiae*, *Escherichia coli*, and *Streptococcus uberis* [10], making the treatment approach complex.

According to the type of clinical manifestation, bovine mastitis occurs in subclinical and clinical forms [11], ranging from mild, moderate, to severe cases [12]. Of the two forms of mastitis, clinical mastitis manifests with visible changes of the milk as well as clinical signs of infection and inflammation, identified by visual investigation. On the other hand, subclinical mastitis (SCM) is difficult to diagnose as the cow appears healthy, while the udder and milk show no visible changes [11]. However, an increased number of somatic cells and the presence of the causative agent are used as the means of detecting SCM. Reported as a more prevalent form [3], SCM is associated with higher losses compared to clinical mastitis [13].

The main treatment of bovine mastitis still relies on antibiotic administration [14]. However, its efficacy is decreasing because of growing drug resistance in bacteria, being considered as a leading global health problem [15]. We are currently facing a rapid global spread of resistance, with the estimation that antimicrobial resistance (AMR) is responsible for over 30,000 deaths per year in the EU [16] and 700,000 deaths per year globally, with a projection of causing millions of deaths [6,17]. In the EU alone, it is estimated that AMR costs EUR 1.5 billion annually in healthcare and productivity losses [18]. Hence, we need to raise awareness of including a multisectoral One Health approach in tackling complex problems such as AMR in order to attain optimal health for people, animals, and the environment [19]. Moreover, antibiotic residues in food and the environment have affected consumers’ concern, thus emphasizing the importance of the reduction of antibiotic use for treating bacterial infections in animals [11,20]. In addition, long-term antibiotic treatment may be required, with a significant economic impact in terms of lost milk production and the cost of the antibiotic [21,22].

Considering the facts mentioned above, the treatment of mastitis is recognized as one of the greatest challenges in recent times. Thus, this work aimed to provide a review of the literature data regarding the possible solutions recognized as alternative treatment approaches to existing conventional bovine mastitis therapy. The availability of vast amount of scientific data may support future research in the study, development, and production of new, effective pharmaceutical formulations with higher efficiency against resistant pathogens.

## 2. Conventional Strategies of Control and Treatment of Bovine Mastitis

### 2.1. Biosecurity

The prevention of infectious diseases is important for animal health, welfare, and production efficiency [23,24,25]. Although this risk can never be totally avoided, it can at least be minimized by the implementation of preventive and control strategies based on improved therapeutic protolocols, hygiene, biosecurity strategies, etc. [5,26]. The National Mastitis Council developed a “5-Point Plan” mastitis control program where numerous approaches for decreasing mastitis incidence are summarized [5,27].

When it comes to prevention, biosecurity measures minimize the risk of new pathogens entering a farm, their transmission within the farm [28,29], and antibiotic usage [25]. Researchers are trying to develop and implement biosecurity tools. Among others, the BIOCHECK CATTLE^®^ protocol for dairy cattle has been developed and used for the biosecurity assessment [30] of farms in Belgium, Kyrgyzstan, North Macedonia, Portugal, and Romania, with a wide variation of biosecurity levels across the farms [31]. Even though the type of biosecurity measures might differ to a large degree between the farming systems [30,32], using this tool, implementation of biosecurity on cattle farms can be assessed in a standardized and reproducible manner [30].

Although the focus on biosecurity as a preventive tool in livestock herds has increased [33], studies demonstrated that most cattle farmers hardly implement all adequate biosecurity measures [34,35,36]. However, protective measures, especially when performed properly, are not always practical and cost time and money, which is important for stakeholders [37,38]. Therefore, the problem of mastitis still requires a wide application of antibiotics for prevention and treatment of mastitis. Probably, further education of farmers on proper implementation of biosecurity measures could improve preventive strategies of bovine mastitis. This way of mastitis management could decrease antibiotic use in prevention and treatment of this disease.

### 2.2. Antibiotic Therapy

Antibiotics are used in intensive livestock production for therapeutic and prophylactic purposes [39], with an estimation that more than 50% of all antimicrobials are used in veterinary medicine globally [15,40]. Van Boeckel et al. [41] projected that antimicrobial consumption will rise by 67% by 2030 in food-producing animals, while the expected rise according to Tiseo et al. [42] is reported to be 11.5%. Bovine mastitis is the most common indication for the use of antibiotics in dairy production [43]. The administration of antibiotics can be performed by local application of intramammary preparations, as well as the systemic application of antibiotics [22], while the intramammary route is the most common route of drug administration for bovine mastitis [44]. The most frequently used antibiotics in mastitis therapy are penicillins, sulfonamides, ampicillin, cloxacillin, and aminoglycosides [45,46]. In addition to antibiotic therapy, symptomatic and supportive therapy are of great importance, alleviating local inflammation of the mammary gland, enabling a better effect of antibiotics due to greater perfusion of the mammary gland as well as faster recovery and regeneration of milk production [22,47].

However, antibiotic therapy has led to numerous concerns regarding public health. Namely, antibiotic use in animals leads to the possibility of transmission of resistant microorganisms through the food chain [48,49], as well as through ecosystems [50]. Besides posing threat to public health, the residues of antibiotics in milk can be a problem in the dairy industry, affecting the technology of production of fermented milk products [40]. Additional consequences of inadequate antibiotic use include partly compromised efficacy, reduced food safety, and changes in cow’s milk [40,51]. Moreover, the financial profitability of the antibiotic should be taken into account [21]. During intramammary treatment with antibiotics in lactation, milk has to be discarded and can only be used again after the withdrawal period has expired, which is quite expensive [48,52]. Some of frequently applied antibiotics are considered critically important for human healthcare, representing an additional global concern related to the protection of public health [15]. Moreover, the discovery and development rate of new antibiotic agents is exceed by the current rate of resistance development [53], which is leading to the search for alternatives together with consumers’ demand for antibiotic-free products [54].

### 2.3. Vaccination

Among preventive strategies applied to minimize bovine mastitis, vaccination is taking significant place. Vaccines are common and effective tools for the control of infective diseases [55], but only few vaccines are fully effective in veterinary medicine [56]. The success of vaccination depends on the vaccine formulation, administration route, vaccination quality, and timely vaccination coverage [57,58].

Immunoprophylaxis enables a modern approach to solving mastitis, providing reduction of antibiotics in therapy, thus affecting antibiotic treatment limitations [59]. Mastitis vaccines are gaining more interest as tools in combating AMR, even though scientific papers in the field of vaccination against the mastitis-associated pathogens point to limited success in obtaining significant results [37]. Additionally, limited success is caused by limited range of protection, since a wide range of strains and their different mechanisms of pathogenesis can be present within an individual cow [21].

Most vaccines are designed to target *S. aureus*, *S. uberis*, *S. agalactiae*, and *E. coli* [22,49], while *S. aureus* mastitis has been particularly difficult to control as the most important etiological agent of bovine mastitis, causing over 50% of the reported cases of mastitis [60]. Various approaches to the vaccine developed against mastitis caused by *S. aureus* have been described in a few reports [37,59,61]. Collado et al. [62] reported the efficacy of the *S. uberis* subunit vaccine against bovine mastitis, with significantly reduced clinical signs of mastitis, bacterial count, rectal temperature, and daily milk yield losses.

Evaluation of a commercially available polyvalent vaccine against mastitis, Startvac^®^, containing inactivated *E. coli* J5 strain and inactivated *S. aureus* SP140 strain, demonstrated different results. Piepers et al. [63] reported more potent immune response and elimination of the bacteria from the mammary gland in comparison with nonvaccinated animals, whereas Landin et al. [64] reported no significant differences between vaccinated and unvaccinated groups. Furthermore, Tashakkori et al. [65] pointed out that vaccination with commercially available Startvac^®^ and Mastivac^®^ was not associated with the reduction of clinical mastitis incidence. However, in order to achieve desired results, vaccination on its own will not usually achieve the desired results unless the vaccination programme is part of an integrated control antimicrobial stewardship strategy utilizing a combination of control measures [22,38]. Nevertheless, improvement of national control programs on vaccination will enable implementation of this strategy as a part of an antimicrobial stewardship program.

## 3. Alternative Strategies—Potential Solutions

Given the importance of bovine mastitis as a public health problem, many in vivo and in vitro studies of alternative treatments for mastitis have been conducted. The introduction of new strategies such as nanotherapy; bacteriophage therapy; animal-, plant-, and bacteria-derived antimicrobials; and probiotics, among others, are aiming to replace conventional antibiotic treatment, as well as solving the problem of AMR. The main advantage of the listed non-antibiotic alternatives is the absence of resistance development [10,66]. Potential solutions, which have been promoted through the One Health concept, as well as numerous EU regulations, aim primarily at reducing antibiotic usage by 50% by 2030 and giving preference to alternative approaches before conventional drugs are applied [67].

### 3.1. Nanotherapy

Nanotechnology is an increasingly growing field of the 21st century with a broad range of applications in industry, engineering, electronics, environment, food, medicine, and consumer products [68,69]. By providing new tools and materials at the nanoscale level that are beneficial for public health [4], nanotechnology has enabled the development of novel treatment options. Interest in their application in veterinary medicine has been rapidly growing, even though scientific data of veterinary nanomedicine are still relatively new and scarce. Recently, nanotechnology has been started to be applied in veterinary medicine in prevention, diagnosis, and therapy. The different types of nanomaterials are also being used for animal breeding, reproduction, and nutrition, and as disinfectants.

Taking into consideration all limitations of antibiotic-based therapy, nanotherapy, by replacing commonly used antibiotics, minimizes the problem of AMR, as well as the problem of drug residues. By this way, nanotherapy has an economic impact, minimizing the amounts of discarded milk and the number of permanently removed cows in dairy herds [70,71,72]. Nanoparticles may serve as potential delivery systems that deliver drugs directly into the target cells [4], enabling the use of very low doses and decreasing the amount of the used drug and withdrawal time in farm animals, which leads to the reduction of cost and side effects [70,71]. Furthermore, these nanoparticles display important biopharmaceutical advantages such as higher intracellular uptake than other conventional forms of drug delivery systems, increasing the accumulation and the retention time of the drug, improving the antibacterial activity, decreasing AMR, and inhibiting the biofilm formation [73]. As an example, nitric-oxide-releasing polymeric particles as a delivery system could be used to combat bovine-mastitis-associated bacteria [74].

According to the literature data, nanoparticles such as silver [72,75], copper nanoparticles [72,75], zinc oxide nanoparticles [76], nanogels [4], and chitosan nanoparticles [77,78] have been reported to show positive results in bovine mastitis management. Such therapeutic techniques are gaining popularity as tools for managing *S. aureus* mastitis due to the antimicrobial activity, including antibiotic-resistant strains and antibiofim activity [4]. The synergistic effect of silver nanoparticles and antibiotics was also evaluated, and a successful combination was obtained using antibiotics, such as erythromycin, in combination with silver nanoparticles against *S. aureus* [20,60]. Considering all mentioned above, veterinary nanomedicine as an innovative approach could play a significant role in the improvement of animal health, welfare, and production [79]. Hence, nanotherapy has a huge potential in the control of the bovine mastitis.

### 3.2. Bacteriophage Therapy

Bacteriophages are defined as viruses that have the ability to infect and replicate inside bacteria, suppressing their proliferation [80,81]. The main advantage of bacteriophages as alternative antibiotics is their specificity. Phages target only the pathogens of interest without affecting the host’s microbiota [82,83]. However, their specificity is also their main limitation [82], due to the fact that a single bacteriophage will only work on a limited number of bacterial strains, meaning that various different phages are needed to treat all possible bacteria associated with infection [84]. Compared to antibiotics, phages multiply in the host cell while the specific host is present, while concentration of antibiotics decreases. Moreover, after solving infection, phages are degraded, while antibiotics can persist in nature for a long time [84]. In addition, unlike antibiotics or vaccines, phages can be easily isolated from the environment, leading to shorter product development time and reduced production costs [85]. Besides being very specific, environmentally friendly, having good biological safety, as well as ability to evolve and to multiply at the site of infections [53,86,87], phages are also praised for their low probability of resistance development [85]. Moreover, bacteriophages also have shown the potential for vaccine developments [22].

Several potential candidates have been identified for bacteriophage therapy against bovine-mastitis-associated *S. aureus* [81,88,89,90], *S. agalactiae* [91], *E. coli* [80,92], *S. uberis* [93], *K. pneumoniae* [94], and *K. oxytoca* [87]. Moreover, the bactericidal effect of phage achieved by affecting bacterial essential cellular processes [85] is making them valuable antibacterial agents with effectiveness against sensitive as well as resistant bacteria [83]. Furthermore, phage therapy will probably not completely replace conventional antibiotics, but it could be employed as an additional treatment option [82], since phages can be used alone, as cocktails, or synergistically with other antimicrobials [95]. Even though bacteriophages can be considered as potential candidates for reducing antibiotic use in livestock production and increasing animal productivity, limitations in their usage in terms of environmental stability require storage and certain special conditions, which limit their usage [66].

### 3.3. Phytotherapy

Phytotherapy is among the most promising and widely used alternative options in the prevention and treatment of different infections in livestock [96,97]. Plants are widely applied due to therapeutic efficacy, low risk of adverse effects, low manufacturing costs, reduced resistance, and low drug residues in animal products and the environment [11,98,99,100]. The antimicrobial, anti-inflammatory, antioxidant, and immunomodulatory efficacy of medicinal plants have been highlighted in numerous studies over the last few years [101,102,103,104]. Furthermore, the multi-target mode of action of plants might be a useful tool in controlling multi-drug resistance in animal pathogens [97,101].

Biological diversity of plants offers an endless supply of new candidates with potential as therapeutic agents [97]. Almost 50% of the main pharmaceuticals available today are derived from natural resources [101]. Moreover, phytotherapy is often utilized in combination with conventional treatment protocols [101,105]. A review paper by Cheesman et al. [106] provides insight into the possibility and advantages of synergistic therapy, highlighting that medical plant–antibiotic combinations not only enhance the antimicrobial effect but also can act as resistance-modifying agents, in addition to other benefits.

Scientific data have reported the effectiveness of many plant-derived compounds in the treatment of mastitis, where essential oils (EOs) such as aromatic oily liquids obtained from plant materials are employed in phytotherapy for a long period of time [96,107]. Physiological and therapeutic benefits displayed by EOs along with GRAS (generally recognized as safe) status provide their wide range of application in veterinary medicine [108]. Due to antimicrobial and antibiofilm activities [109] EOs are being considered as a subject of interest to the scientific community in the development of novel therapeutic options. The antimicrobial activity of several different EOs on common bovine mastitis pathogens was the subject of research in different in vitro studies [105,110,111,112,113,114,115,116,117,118], while some authors focused on their antibiofilm effect [118,119,120].

While there have been many in vitro studies on the efficacy of EOs relating the potential use in bovine mastitis treatment, only few of them targeted in vivo efficacy. Pharmaceutical formulations based on plant extracts and EOs under the form of a spray, gel, ointment, or infusion have been developed [99,114,121,122,123,124,125,126], and different results of clinical and bacteriological cures were reported. In addition, the commercial product Phyto-Mast^®^ composed of extracts of *Thymus vulgaris*, *Gaultheria procumbens*, *Glycyrrhiza uralensis*, *Angelica sinensis*, and vitamin E, in the treatment of clinical mastitis in dairy cows, did not result in a resolution of clinical mastitis, nor a bacteriological cure [121]. However, Mullen et al. [122] reported that therapy effect of Cinnatube^®^ and Phyto-Mast^®^ was similar to conventional therapy, without an irritating effect on the udder. Furthermore, Tomanic et al. [126] suggested the possible use of an EO-based pharmaceutical formulation (Phyto-Bomat^®^) consisting of *Thymus vulgaris*, *Thymus serpyllum*, *Origanum vulgare*, *and Satureja montana* as a component in mastitis control programs due to the resolution of symptoms post-treatment, as well as the prevention of the development of clinical mastitis in cases with subclinical mastitis.

As mentioned earlier, intramammary applied antibiotics consequently lead to deposition of residues in milk, affecting food safety, as well as economic profitability [127]. However, Kovačević et al. [52] reported minimal milk residues of thymol and carvacrol, two major chemical compounds of Phyto-Bomat^®^ pharmaceutical formulation, which after 24 h decreased to the same level as before application. Moreover, in the study conducted by McPhee et al. [128] thymol residues in milk samples of goats were only detecTable 12 h after treatment with Phyto-Mast^®^.

The literature data suggest that the limitation of EO application could be due to the instability, biodegradability, and low solubility in certain solutions, pointing out that the development of delivery systems, such as metal nanoparticles, could improve previously mentioned EO shortcomings [4]. Moreover, pharmacoeconomic analysis was applied in veterinary medicine for the first time in order to assess the clinical and economic value of the EO-based alternative (Phyto-Bomat), highlighting economic benefits (savings) for farmers associated with the use of this treatment, with a focus on subclinical mastitis, since it contributes to most of the financial losses [129]. In line with the aforementioned, phytotherapy has a great potential in the management of bovine mastitis, especially EO-based formulations.

### 3.4. Homeopathy

Although quite controversial, the use of homeopathy in food-producing animals is evidence-based [130,131], even though a small number of scientific studies on the evaluation of the efficacy of homeopathy have been conducted so far. As an alternative treatment method, homeopathy is enjoying increasing popularity, particularly on organic farms, as a way of combating AMR [131]. Homeopathy, which is based on a holistic approach of stimulating the animal’s immune system [132], besides clinical signs and the pathogen agents, considers also behaviour, constitution, and environmental factors [130].

However, references to homeopathy are limited concerning bovine mastitis. While some studies found negative or no effects of homeopathic therapy for mastitis [133,134], Mimoune et al. [135] reported an encouraging clinical success rate of tested homeopathic preparation in the treatment of this disease. Furthermore, in India [136], the testing of homeopathic combination medicine was effective and economical in the management of mastitis in lactating dairy cows. According to Zeise and Fritz [137], homeotherapy in combination with antibiotics could be one of the options for successful control of bovine mastitis. Regardless, homeopathy offers some great advantages—it does not induce an allergic reaction or other side effects, no residue problems, no withdrawal period for the product, and no environmental pollution. In addition, the remedies are relatively cheap [132,136]. On the other hand, this alternative treatment can be associated with disadvantages due to their simplified registration, as most homeopathic formulations are present in the market without pharmacological, toxicological, or clinical assessment [133]. Further research in the field of homeopathy is needed in order to prove the efficiency of this alternative approach.

### 3.5. Bacteria-Derived Antimicrobials

Antimicrobial peptides such as bacteriocins are ribosomally synthesized peptids produced by bacteria. Being biologically active against Gram-positive and Gram-negative bacteria, they represent an alternative solution to conventional antibiotics [49,138,139]. Compared to antibiotics, bacteriocins have a narrow spectrum of antimicrobial activity, targeting specific pathogenic organisms [140,141] and efficiency against antibiotic-resistant strains. Moreover, they show antimicrobial activity at lower concentrations than antibiotics [142]. Bacteriocins of lactic acid bacteria demonstrated in vitro inhibitory antimicrobial activity against mastitis-associated Gram-positive organisms such as *S. uberis* and *S. agalactiae* [49,138,139].

A bacteriocin produced by *Lactococcus lactis* known as nisin is reported in the literature data as a therapeutic option due to its efficacy against mastitis-associated pathogens and foodborne pathogens [49,139,143]. Nisin, as well as several other bacteriocins, have generally regarded as safe (GRAS) status, being approved for application as food preservatives [142,144]. Currently, nisin is available in some countries for use in the dairy industry for teat disinfection in the form of wipes (Wipe Out^®^) [49,139]. Furthermore, Bennett et al. [141] demonstrated promising results of bactofencin, nisin, and reuterin in bovine mastitis treatment, being active against multidrug-resistant clinical bovine mastitis isolates, while a study by Hernández-González [144] demonstrated the antimicrobial activity of nisin on biofilm-producing *S. aureus* cultures. Moreover, promising results of the aureocins A70 and A53 combination against *S. aureus*-associated bovine mastitis were reported by Coelho et al. [145]. Some bacteriocins can act synergistically with conventional antibiotics, reducing concentrations, undesirable side effects, and the prevalence of resistant strains [144].

### 3.6. Animal-Derived Antimicrobials

Lactoferrin is a multifunctional glycoprotein found in saliva, tears, bronqueal mucus, colostrum, and milk [21,22], with a broad range of biological activities including antimcrobial, antiinflammatory, immunomodulatory, anticatabolic, and antioxidative effects [146,147]. Several in vitro studies reported an antibacterial effect against some major mastitis-causing pathogens [148,149,150], showing not only bacteriostatic but also bactericidal and antifungal activities [151]. Moreover, it can be effective as a preventive or therapeutic option for bovine mastitis [151].

Milk contains not only lactoferrin but also antibacterial proteins such as lysozyme, immunoglobulins, lactoperoxidase, and β-defensin with a mechanism of action consisting of inactivation of bacteria, prevention of bacterial adherence to mammary tissue, and neutralization of toxins [151]. Lysozyme with a hydrolyzing effect on the essential bacterial cell component peptidoglycan leading to its lysis [49,152] was used successfully in increasing antibiotic efficacy against *S. uberis* and *S. dysgalactiae* associated with bovine mastitis [49]. Chaneton et al. [153] suggests that β-lactoglobulin and lactoferrin may complement each other against bacterial infection, due to the different effects on different bacteria, which expend their spectrum of antimicrobial activity [21]. It was reported that lactoferrin, lysozyme, and other peptids as potential non-antibiotic antimicrobial agents for the treatment and prevention of bovine mastitis can be used in combination with antibiotics [147,149,154].

When it comes to other animal-derived treatment options for mastitis, propolis is one of the most widely known and used natural products. As a natural resinous mixture produced by honeybees from substances collected from different plants [155,156], propolis is enriched with bees’ salivary and enzymatic secretions [157]. Furthermore, propolis has a complex chemical composition, with identified compounds such as polyphenols, terpenoids, steroids, and amino acids [155]. In addition, propolis has several biological activities such as antiviral, antibacterial, antifungal, immunostimulatory, antioxidant, imunomodulatory, hepatoprotective, and anti-inflammatory effects [155,158,159], leading to its application in the development of products for use in human and animal health [158].

In the study conducted in Croatia, it is described that propolis could be used in the development of an intramammary pharmaceutical formulation for the prevention and treatment of bovine mastitis during lactation [160,161]. Moreover, this innovative intramammary formulation of a non-alcoholic solution of propolis showed antibacterial, immunostimulatory, antioxidant, and anti-inflammatory activity, with the potential to become an alternative to conventional therapy [160,162]. Furthermore, Hegazi et al. [155] confirmed the efficacy of propolis as an antibacterial agent against bacterial strains isolated from mastitis, where propolis exhibited significant antimicrobial activity against ≈41% of the isolated pathogens, being more effective against Gram-positive bacteria than Gram-negative bacteria [155]. These results are in accordance with the work of Bačić et al. [161], where an in vitro tested propolis formulation also exhibited higher antimicrobial activity against Gram-positive bacteria than Gram-negative bacteria. Among all animal-derived antimicrobials, the clinical efficiency of pharmaceutical formulation based on propolis in the prevention and treatment of bovine mastitis can lead to the reduction of antibiotics usage and AMR in livestock production.

### 3.7. Other Alternatives

Other alternatives have included the use of probiotics [163,164], zeolites [165,166], cytokines [167,168,169,170], ozone [171,172,173], and aloe vera [174,175]. Zeolites are natural, microporous, three-dimensional crystalline materials with well-defined channels and cavities and various technological applications in veterinary medicine [176,177]. Moreover, a widely used zeolite is known as clinoptilolite, which has been proven as safe for veterinary use [178], acting as an antibacterial, antiviral, antioxidant, anti-diarrheic, and growth-promoting agent [165,166,178,179]. Đuričić et al. [165], studying dietary vibroactivated and micronised clinoptilolite, found out its effect on reducing the incidence of intramammary infections, while Ural [180] reported the efficacy of supplementation with 3% clinoptilolite in dairy cows in terms of milk yield and somatic cell count.

Probiotics are gaining more attention as an interesting alternative for the prevention or treatment of bovine mastitis [181,182]. Pellegrino et al. [183] demonstrated that lactic acid bacteria, as one of the components of the indigenous microbiota of the teat canal, could be used as candidates in mastitis prevention, while Klostermann et al. [184] reported that intramammary administration of a live culture of *Lactococcus lactis* in some cases may be as effective as antibiotic treatment. Researchers worldwide are trying to develop probiotic-based formulations for the prevention and treatment of mastitis, since they have shown great potential in this field.

In conclusion, much of the reviewed data have shown the opportunity of exploiting novel therapeutic approaches in overcoming the limitations of conventional antibiotic-based therapies. The reviewed alternative options will probably not completely replace conventional antibiotics, but they could be employed as an additional or supportive option in the treatment of infections. When it comes to EO-based pharmaceutical alternatives, Phyto-Mast and Cinnatube have great potential in achieving similar results as conventional therapy. Furthermore, Phyto-Bomat could be an important part of the mastitis control program, as a treatment approach in subclinical mastitis, and a supportive option in clinical mastitis. In addition, phytotherapy as an alternative approach has shown good results against resistant strains and biofilm formation. Moreover, nanotherapy and use of a propolis-based formulation have shown significant results in clinical studies of bovine mastitis therapy. As an important part of antimicrobial stewardship, the use of antimicrobial alternatives together with other strategies such as vaccination and biosecurity measures could help us to enhance public health by decreasing AMR.

## Data Availability

Not applicable.
